# Intergovernmental or fully independent? Designing a scientific panel on evidence for action against antimicrobial resistance

**DOI:** 10.1371/journal.pgph.0004039

**Published:** 2025-01-03

**Authors:** Arne Ruckert, Steven J. Hoffman, Julia Bishop, Susan Rogers Van Katwyk, Patrick Fafard, Mathieu J. P. Poirier

**Affiliations:** 1 Global Strategy Lab, Dahdaleh Institute for Global Health Research, York University, Toronto, Ontario, Canada; 2 School of Global Health, Faculty of Health, York University, Toronto, Ontario, Canada; 3 Graduate School of Public and International Affairs, University of Ottawa, Ottawa, Ontario, Canada; Washington University School of Medicine, UNITED STATES OF AMERICA

## Abstract

Effective global action against antimicrobial resistance (AMR) relies on the successful synthesis and translation of rigorous scientific evidence into policy and practice. Despite a call in 2019 by the Interagency Coordination Group on AMR to establish a policy-science interface, and the reaffirmation to establish a scientific panel in the 2024 Political Declaration on Antimicrobial Resistance, no authoritative entity currently exists that synthesizes the scientific evidence on AMR and outlines policy options based on the best scientific insight. A Scientific Panel on Evidence for Action against AMR (SPEA) could address this gap, as well as contribute to additional governance gaps in the space of AMR, by facilitating better global coordination and cooperation; establishing real-time evidence to guide policy actions; and monitoring progress towards any globally agreed upon AMR goals and targets. In this essay, we argue that SPEA has the potential to fulfill several governance functions, and we explore two design options for such a scientific panel to promote equitable and evidence-informed policy implementation. We first reflect on how the successes and failures of the Intergovernmental Panel on Climate Change (IPCC) should inform the SPEA. Building on these lessons, we then highlight the key functions of the SPEA, before proposing two models for how it could function in the context of the existing global governance of AMR. Finally, we reflect on the challenges inherent to each proposed governance model. The recent reaffirmation by the United Nations General Assembly to establish a scientific panel in the area of AMR represents a critical opportunity to enhance global AMR governance, promote evidence-based policy implementation, and foster international cooperation in combatting AMR.

## Introduction

In 2019, the United Nations Interagency Coordination Group for the Global Governance of Antimicrobial Resistance (AMR) called for the establishment of three bodies to contribute to the global governance of AMR [1]. Two of these bodies have already been created: the Global Leaders Group on AMR (GLG), which aims to promote sustained political action on antimicrobial resistance [2]; and the AMR Multi-Stakeholder Partnership Platform (MSPP), which aims to catalyze a global movement for action against AMR by fostering cooperation between a diverse range of stakeholders across the One Health spectrum [3]. The third body, the Independent Panel on Evidence for Action against Antimicrobial Resistance, has not been established yet, despite the development of draft Terms of Reference and concrete guidance by the Interagency Coordination Group for the Global Governance of AMR about what a scientific panel on AMR could look like, as well as the installment of a WHO advisory group in 2020 to ensure progress on setting up such a scientific panel [1].

This lack of progress is problematic for a variety of reasons. A scientific panel was meant to inform the work of the GLG and MSPP by building an evidence base for policymakers and AMR researchers to allow for the prioritization and arbitration of effective AMR interventions. In addition, it was supposed to help coordinate an effective international policy response, monitor, and provide Member States with regular reviews of the science and evidence related to AMR. This includes stocktaking of existing AMR initiatives and their impacts, and recommending policy options for adaptation and mitigation [4]. The importance of building such a panel was reaffirmed in the Political Declaration on Antimicrobial Resistance signed during the High-level Meeting on Antimicrobial Resistance at the 2024 United Nations General Assembly which specifically calls for the establishment of “an independent panel for evidence for action against antimicrobial resistance in 2025 to facilitate the generation and use of multisectoral, scientific evidence to support Member States in efforts to tackle antimicrobial resistance, making use of existing resources and avoiding duplication of on-going efforts” [5].

With significant financial commitments from the United Kingdom and other countries already announced towards establishing a science-policy interface for AMR, the international community needs to reflect on how to move forward in building a scientific panel on evidence for action against AMR (SPEA) [6]. Although the term ‘independent panel’ has been widely used in global discussions about establishing a science-policy interface for AMR, we use the term scientific panel as an umbrella category to capture both independent and intergovernmental panel approaches. In this essay, we argue that a scientific panel has the potential to fulfill several crucial functions in the AMR global governance ecosystem and explore different design options for such a panel to promote equitable and evidence-informed policy implementation. We first reflect on how the successes and failures of the Intergovernmental Panel on Climate Change (IPCC) can inform the SPEA. Building on these lessons, we then highlight the key functions of the SPEA, before proposing two models for how it could function in the context of the existing global governance of AMR.

## Lessons from the IPCC for the design of a SPEA

Building on a recently published white paper [7], and previous research we conducted on public health advice to national governments [8], as well as global governance design principles [9], we have identified four key insights that can be derived from the IPCC to inform the design of a SPEA: the importance of legitimacy and credibility; having clear zones of independence to reduce the political and commercial influence on scientific processes; accelerating evidence synthesis and reporting timelines; and the need for more cost-effective and efficient review systems.

First, a SPEA must have legitimacy and credibility to ensure Member State engagement and political buy-in by all AMR stakeholders. A sense of ownership by Member States and meaningful engagement of all relevant AMR OH stakeholders can contribute to generating such legitimacy. Regardless of its governance model, a SPEA must be inclusively designed with equal input from high-income and low- and middle-income countries (LMICs), ensuring that the policy priorities of the Global North are not dominating the international AMR policy response. Transparent and inclusive governance processes are key aspects of establishing SPEA’s legitimacy and credibility.

Second, some have contested the IPCC’s legitimacy due to the prominent role of governments in its workflow, including the role of politically appointed reviewers in the peer-review process for major IPCC reports [10]. Thus, a SPEA needs to establish clearer zones of independence than exist in the IPCC between its political and policy functions and its scientific evidence synthesis operations. This should include building and maintaining a firewall between the political selection process of scientific experts and their role in the evidence review process, as well as ensuring protections to prevent undue corporate influence or policy capture by commercial actors. This will require a transparent process for selecting panel members and reviewers based on their expertise, experience, and independence from political or commercial interests. This process should further involve nominations from a diverse set of One Health stakeholders, including governments, academia, civil society, and industry, followed by thorough vetting to ensure as much impartiality as possible, as initially proposed in the draft terms of reference for IPEA [11].

Third, the IPCC’s assessment reports typically take several years to produce, from the initial scoping phase to final approval and publication [12]. This issue is of particular concern in the context of AMR evidence generation, where our understanding of interventions to address AMR is still limited but has been developing rapidly in recent years. The sheer volume of new AMR evidence makes it increasingly challenging for policymakers to remain up-to-date, making a rapid evidence synthesis process a necessary aspect of global AMR governance [13]. The rapidly evolving nature of AMR evidence also implies a need for faster review completion rates. This could be achieved by creating living systematic reviews, building on existing, scientifically validated platforms that are already providing high-quality AMR data. For example, the GRAM project and Global PPS survey offer critical insights into human health [14], while resources like ResistanceBank.org [15] and the Global Burden of Animal Diseases (GBAD) address AMR and disease prevalence in animals [16]. Finally, a new initiative by the Wellcome Trust, the Evidence Synthesis Infrastructure Collaborative, aims to create a common data infrastructure integrating artificial intelligence (AI)-based tools that can help evidence synthesis experts extract insights or produce summaries more quickly [17]. Living systematic reviews should build on existing data infrastructure, be maintained as an accessible global public good, and focus on specific policymaker needs for high-quality evidence. All data and evidence synthesis by SPEA should include a commitment to open data principles, ensuring that the scientific community can access any synthesized data to allow open assessment of AMR and antimicrobial use trends.

Fourth, to facilitate rapid review processes, less expensive and bureaucratic structures should be used, including building on existing research and evidence networks focused on AMR. This should include exploring ways to integrate existing knowledge translation structures and following established systematic review protocols, such as the rigorous standards established by Cochrane, the Campbell Collaboration, or the Collaboration for Environmental Evidence. Where possible, SPEA should align its efforts with other complementary initiatives such as the Global Coalition for SDG Syntheses and the Global Alliance for Living Evidence (GALE), to mitigate the potential for duplicated efforts and promote synergies in attaining AMR-related global goals such as the Sustainable Development Goals.

## The SPEA will support better global governance of AMR

With many AMR coordination challenges remaining, one of the primary objectives of a SPEA should be to encourage international cooperation in building a One Health evidence base to address AMR, bringing together experts from diverse fields such as human, animal, and plant health, agriculture, and social sciences. Effective monitoring and surveillance are crucial to understanding and addressing AMR, both in terms of building international AMR surveillance systems, as well as evaluating AMR policy implementation. The AMR community needs a structure that can facilitate stocktaking to know if we are collectively on track to meet internationally agreed upon AMR goals or targets. Establishing global (political) goals and country- and sector-specific (technical) targets for AMR is central to guiding our collective efforts, promoting accountability, and driving coordinated action among stakeholders. Once such AMR goals or targets have been established globally, SPEA can become the recognized arbiter of progress towards achieving them [18]. Recent examples of proposed goals and targets include the 1-10-100 Unifying Goals for AMR proposed by the Bellagio Group for Accelerating AMR Action [19], as well as the 10-20-30 targets proposed as part of *The Lancet Series on Antimicrobial Resistance* [20] (see Box 1).

**Box 1: Proposed goals and targets for AMR**

Effective management of AMR hinges on the establishment of clear, actionable goals and targets that can guide and measure global efforts. **The 1-10-100 Unifying Goals** were proposed by the Bellagio Group for Accelerating AMR Action, a collective of leading researchers and policymakers in the field. The 1-10-100 framework highlights the need for using a One Health approach to safeguard health and the environment, aims to save 10 million lives through better infection prevention and treatment and facilitate 100% sustainable access to antimicrobials by 2040. **The 10-20-30 targets** were featured in The Lancet Series on Antimicrobial Resistance, which highlights the critical threat AMR poses to modern medicine and the urgent need for sustainable access to effective antibiotics. These targets were crafted to catalyze progress in the global fight against AMR by setting clear and measurable objectives. The 10-20-30 framework aims to reduce mortality from AMR by 10%, reduce inappropriate antibiotic use in humans by 20%, and decrease inappropriate antibiotic use in animals by 30% by 2030. Although the Political Declaration on Antimicrobial Resistance committed to reduce global deaths associated with bacterial antimicrobial resistance by 10 per cent by 2030 against the 2019 baseline of 4.95 million deaths, these more comprehensive frameworks could be used to structure high-level political goals to galvanize the global effort to address AMR.

A central aspect of SPEA’s set-up must be the ability to create real-time evidence guiding policy actions that acknowledge that there is no ‘silver bullet’ or ‘one size fits all’ in AMR. Recognizing this means that policy options must always be tailored to specific ecological situations and socio-economic contexts, as well as the health challenges of diverse populations [21]. In addition, the SPEA’s recommendations must encompass a wide range of topical areas, including access to antimicrobials and their stewardship; surveillance systems; regulatory frameworks; and governance considerations, with the overarching goal of mitigating the impact of AMR on global health. As many urgent evidence gaps persist, with much of the evidence on AMR being contradictory or of poor quality, it remains challenging for decision-makers to weigh the merits of proposed AMR interventions. For example, evidence on AMR in populations with increased exposure or heightened vulnerabilities to AMR is currently limited, including for gender-diverse populations, rural/remote populations, or migrants and refugees [22]. A central aspect of SPEA must therefore be ensuring that equity considerations are meaningfully integrated into all systematic reviews, at minimum by following the PRISMA equity extension for systematic review reporting guidelines [23], as well as applying the Campbell Collaboration equity checklist for systematic reviews in all operations of SPEA [24].

Given its purpose to produce global public goods, a SPEA could serve an important function in supporting LMICs to address AMR [7]. People in LMICs face a disproportionate AMR burden due to higher rates of infectious diseases and limited access to healthcare and other essential services [25]. Since the drivers of AMR are not the same across socio-economic settings, a SPEA should ensure that evidence syntheses consider cost-effective strategies tailored to resource-constrained settings and promote equitable and prevention-focused social infrastructure interventions, including access to water, sanitation, and hygiene (WASH), expanded vaccination programs, and facilitating better access to antimicrobials [7].

Finally, a SPEA can also play a role in producing globally credible evidence and AMR monitoring needed for advocacy and public awareness building in the global space of AMR. The climate activism we have seen emerge over the last decade would be unthinkable without the evidence base that IPCC has produced, and the impact it has had on popular perception on climate change [26]. Similarly, we can expect SPEA to not only transform how policymakers engage with evidence to guide AMR interventions but to also shape public perception and actions around AMR.

## Two models for the SPEA in AMR global governance

**Fig 1:**
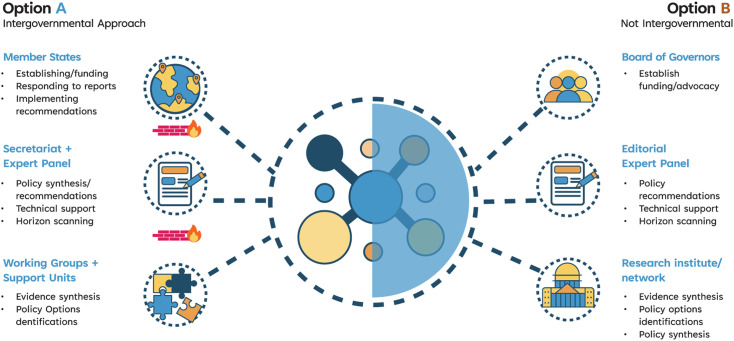
Two models for the governance of SPEA.

Looking ahead, there are two models for how the SPEA could be designed to function in the global governance of AMR (see Fig 1): an intergovernmental approach or a fully independent panel approach. The intergovernmental approach is similar to that of the IPCC where Member States would initially fund the SPEA, select an Expert Panel and Working Groups, and establish a Secretariat to facilitate implementation. However, Working Groups and their associated Technical Support Units would need to be protected by institutional firewalls between the Member States and the Expert Panel and Secretariat, as well as from external political or economic interests and interference [7]. In this intergovernmental model, Member States would be responsible for establishing and funding the SPEA, as well as responding to reports and implementing recommendations. The thematic Working Groups would provide an initial assessment of the scientific literature on AMR, building the much-needed evidence base on AMR, while the Expert Panel would identify specific review needs via horizon scanning and synthesize the policy options developed by the Working Groups into recommendations for government decision-makers. The Expert Panel would ideally be composed of AMR experts who are not selected by governments but rather by the Secretariat based on their specific AMR expertise and a diversity of perspectives across all relevant One Health disciplines. In this model, the Secretariat would be responsible for liaising directly with Member States, coordinating the work of the Expert Panel, and contributing to the dissemination of report. The main advantage of such a model would be the immediate financial and political support it would receive from its Member States, which would build a sense of ownership and make the uptake of the SPEA’s policy recommendations more likely. However, the potential for political interference remains a risk of this model, as does the need for widespread political agreement between countries which could slow down the SPEA’s establishment.

Alternatively, a second model would create a fully independent panel through independent funding or a coalition of committed countries and funders, gaining credibility and legitimacy through a Board of Governors comprised of high-level AMR leaders and scientific experts [7]. Such an independent coalition of AMR leaders and experts would serve an advocacy role to accelerate the adoption of policy recommendations by relevant stakeholders. Under this model, evidence would be curated, and policy options developed by an independent network of experts hosted at relevant research institutions in the space of AMR. Such an AMR evidence collaboration could be built on already existing structures, such as WHO, WOAH, FAO, and UNEP Collaborating and Resource Centres in AMR or relevant Cochrane Collaboration Centres. In this model, the Editorial Expert Panel would be responsible for the coordination of activities and development of specific policy recommendations and communicating findings to both the AMR leaders, as well as state and non-state implementers of recommendations. Members of that panel should be selected based on expertise and equity criteria, limiting political influence over the selection process [11]. This represents one of the main advantages of this model, as does the ability to start building a SPEA even in the absence of international agreement among governments and international organizations about the need for SPEA. However, without direct government participation and coordination, there is a higher risk that the SPEA could fail to secure sustainable dedicated funding, and its recommendations could lack credibility with, and relevance to the needs of UN Member States, and therefore fail to raise AMR’s political profile or successfully catalyze policy adoption. Similar concerns with political and commercial interference as outlined in the first model would exist and need to be addressed, as would the need for coordination with existing structures within Quadripartite organizations to avoid duplication or overlapping mandates. While the Quadripartite has developed capacity for providing technical guidance to Member States, its ability to efficiently synthesize the breadth of AMR evidence is circumscribed by broader institutional constraints. These could be overcome by an independent institution with dedicated resourcing and a mandate to regularly update the global AMR research agenda [27].

Several factors have led to delays in the establishment of a SPEA, including the fragmented nature of global AMR governance, the inability to mobilize the required resources to operate the SPEA, and a focus on acute global health threats during the COVID-19 pandemic. The 2024 Political Declaration’s call for the establishment of an independent panel in 2025 represents an opportunity to overcome many of these barriers, as it signals strong political backing via the UN and its Member States, and directly invites members of the Quadripartite to establish a SPEA [5]. The Political Declaration also commits to an implementation timeline and highlights the importance of making use of existing resources to avoid duplication of ongoing efforts. This framing could help address concerns about fragmentation by clearly outlining the SPEA’s role in facilitating the generation and use of multisectoral, scientific evidence, while also safeguarding existing AMR advisory bodies, such as WHO’s Strategic and Technical Advisory Group for AMR. This renewed collaborative strategy could overcome barriers that hindered previous progress, ensuring that the establishment of the SPEA gains the necessary political momentum to elevate AMR as a key global priority.

## Conclusion

Effective global action against AMR relies on the successful synthesis and translation of rigorous scientific evidence into policy and practice. Despite calls from the Interagency Coordination Group to establish a policy-science interface, no authoritative entity currently exists that synthesizes the scientific evidence on AMR and offers policy options based on the best scientific insights. The recommitment to the establishment of a scientific panel expressed in the 2024 Political Declaration on Antimicrobial Resistance represents a unique window of opportunity for Member States to firmly commit to its establishment, providing renewed hope that previous barriers will be overcome. Establishing a SPEA will not only allow governments to draw on empirical evidence on what policies to pursue in mitigating AMR but also help to fulfill crucial governance functions in AMR that currently remain underdeveloped, including: facilitating better global coordination and cooperation; establishing real-time evidence to guide policy actions; monitoring progress towards any globally agreed upon AMR goals and targets; and engaging in advocacy to enhance public awareness surrounding AMR. Effective AMR policymaking relies on the availability of up-to-date, policy-relevant recommendations of policy options for AMR mitigation based on rigorous evidence synthesis, necessitating a SPEA to establish a unified, equitable, and authoritative evidence base on AMR.

## References

[1] Interagency Coordination Group on Antimicrobial Resistance (ICGA). No time to wait: securing the future from drug resistant infections. New York: United Nations; 2019. Available from: https://www.who.int/docs/default-source/documents/no-time-to-wait-securing-the-future-from-drug-resistant-infections-en.pdf?sfvrsn=5b424d7_6

[2] Global Leaders Group on Antimicrobial Resistance. Towards specific commitments and action in the response to antimicrobial resistance. 2024 Mar. Available from: https://www.amrleaders.org/resources/m/item/glg-report

[3] FAO. The platform | Antimicrobial Resistance | Food and Agriculture Organization of the United Nations. 2024 [cited 17 May 2024]. Available from: https://www.fao.org/antimicrobial-resistance/quadripartite/the-platform/en/

[4] FAO, UNEP, WHO, WOAH. Call for actionable steps in response to the rising threat of antimicrobial resistance (AMR). Rome: FAO; 2024. p. 13. Available from: https://openknowledge.fao.org/handle/20.500.14283/cd0529en

[5] United Nations. Political declaration of the high-level meeting on antimicrobial resistance. New York: United Nations; 2024. p. 15.

[6] Dall C. UK announces funding to boost global fight against antimicrobial resistance | CIDRAP. 16 May 2024 [cited 10 Jun 2024]. Available from: https://www.cidrap.umn.edu/antimicrobial-stewardship/uk-announces-funding-boost-global-fight-against-antimicrobial-resistance

[7] Ruckert A, Bishop J, Poirier MJ. Designing an independent panel on evidence for action against AMR. Toronto: Global Strategy Lab; 2024. p. 7. Available from: https://amrpolicy.org/wp-content/uploads/sites/2/2024/06/GSLPolicyBrief_IPEA-2024-06-13.pdf

[8] Cassola A, Fafard P, Nagi R, Hoffman SJ. Tensions and opportunities in the roles of senior public health officials in Canada: a qualitative study. Health Policy. 2022;126(10):988–95. doi: 10.1016/j.healthpol.2022.07.009 36002358 PMC9296232

[9] Weldon I, Rogers Van Katwyk S, Burci GL, Giur D, de Campos TC, Eccleston-Turner M, et al. Governing global antimicrobial resistance: 6 key lessons from the Paris climate agreement. Am J Public Health. 2022;112(4):553–7. doi: 10.2105/AJPH.2021.306695 35319963 PMC8961837

[10] Shaw A. Policy relevant scientific information: the co-production of objectivity and relevance in the IPCC. 2005 [cited 17 May 2024]. Available from: https://escholarship.org/uc/item/0d81p739

[11] WHO. Public discussion - draft terms of reference of the independent panel on evidence for action against antimicrobial resistance. 2020. Available from: https://www.who.int/publications/m/item/public-discussion-draft-terms-of-reference-independent-panel-on-evidence-amr

[12] Pollitt H, Mercure J-F, Barker T, Salas P, Scrieciu S. The role of the IPCC in assessing actionable evidence for climate policymaking. NPJ Clim Action. 2024;3(1):11. doi: 10.1038/s44168-023-00094-x

[13] Luz CF, van Niekerk JM, Keizer J, Beerlage-de Jong N, Braakman-Jansen LMA, Stein A, et al. Mapping twenty years of antimicrobial resistance research trends. Artif Intell Med. 2022;123:102216. doi: 10.1016/j.artmed.2021.102216 34998519

[14] GBD 2021 Antimicrobial Resistance Collaborators. Global burden of bacterial antimicrobial resistance 1990-2021: a systematic analysis with forecasts to 2050. Lancet. 2024;404(10459):1199–226. doi: 10.1016/S0140-6736(24)01867-1 39299261

[15] Criscuolo NG, Pires J, Zhao C, Van Boeckel TP. resistancebank.org, an open-access repository for surveys of antimicrobial resistance in animals. Sci Data. 2021;8(1):189. doi: 10.1038/s41597-021-00978-9 34294731 PMC8298417

[16] Gilbert W, Marsh TL, Chaters G, Jemberu WT, Bruce M, Steeneveld W, et al. Quantifying cost of disease in livestock: a new metric for the global burden of animal diseases. Lancet Planet Health. 2024;8(5):e309–17. doi: 10.1016/S2542-5196(24)00047-0 38729670 PMC11636736

[17] Wellcome Trust. Evidence synthesis infrastructure collaborative. 2024. Available from: https://wellcome.org/news/evidence-synthesis-infrastructure-collaborative

[18] Tejpar S, Rogers Van Katwyk S, Wilson L, Hoffman SJ. Taking stock of global commitments on antimicrobial resistance. BMJ Glob Health. 2022;7(5):e008159. doi: 10.1136/bmjgh-2021-008159 35589150 PMC9121412

[19] Rogers Van Katwyk S, Poirier MJP, Chandy SJ, Faure K, Fisher C, Lhermie G, et al. 1-10-100: unifying goals to mobilize global action on antimicrobial resistance. Global Health. 2024;20(1):66. doi: 10.1186/s12992-024-01070-8 39187834 PMC11348599

[20] Mendelson M, Lewnard JA, Sharland M, Cook A, Pouwels KB, Alimi Y, et al. Ensuring progress on sustainable access to effective antibiotics at the 2024 UN general assembly: a target-based approach. Lancet. 2024;403(10443):2551–64. doi: 10.1016/S0140-6736(24)01019-5 38797179

[21] Weldon I, Hoffman SJ. “Fit for purpose?” Assessing the ecological fit of the social institutions that globally govern antimicrobial resistance. Perspect Politics. 2024;1–22. doi: 10.1017/s1537592723002906

[22] Gautron JMC, Tu Thanh G, Barasa V, Voltolina G. Using intersectionality to study gender and antimicrobial resistance in low- and middle-income countries. Health Policy Plan. 2023;38(9):1017–32. doi: 10.1093/heapol/czad054 37599460 PMC10566319

[23] Welch V, Petticrew M, Tugwell P, Moher D, O’Neill J, Waters E, et al. PRISMA-equity 2012 extension: reporting guidelines for systematic reviews with a focus on health equity. PLoS Med. 2012;9(10):e1001333. doi: 10.1371/journal.pmed.1001333 23222917 PMC3484052

[24] The Campbell Collaboration. Equity checklist for systematic review authors. 2009. Available from: https://www.campbellcollaboration.org/images/pdf/plain-language/C2_Equity_Checklist.pdf

[25] Mendelson M, Laxminarayan R, Limmathurotsakul D, Kariuki S, Gyansa-Lutterodt M, Charani E, et al. Antimicrobial resistance and the great divide: inequity in priorities and agendas between the Global North and the Global South threatens global mitigation of antimicrobial resistance. Lancet Glob Health. 2024;12(3):e516–21. doi: 10.1016/S2214-109X(23)00554-5 38278160

[26] Paglia E, Parker C. The intergovernmental panel on climate change: guardian of climate science. In: Boin A, Fahy LA, 't Hart P, editors. Guardians of public value: how public organisations become and remain institutions. Cham: Springer International Publishing; 2021. p. 295–321.

[27] Fieldman T, Mossialos E, Anderson M. Enhancing global insight into AMR spread and generation: prospects and limitations of the WHO and quadripartite research agendas. J Antimicrob Chemother. 2024;79(2):207–10. doi: 10.1093/jac/dkad393 38153237

